# Intravenous iron for anaemia in pregnancy: A quantitative study of acceptability and feasibility of its integration into routine antenatal care practice in Nigeria

**DOI:** 10.1371/journal.pone.0328239

**Published:** 2026-02-04

**Authors:** Opeyemi Rebecca Akinajo, Aduragbemi Banke-Thomas, Kristi Sidney Annerstedt, Lenka Beňová, Yusuf Adetomiwa Adelabu, Nadia Adjoa Sam-Agudu, Bosede Bukola Afolabi

**Affiliations:** 1 Department of Obstetrics and Gynaecology, Lagos University Teaching Hospital, Idi-Araba, Lagos, Nigeria; 2 Department of Global Public Health, Karolinska Institutet, Stockholm, Sweden; 3 Department of Public Health, Institute of Tropical Medicine, Antwerp, Belgium; 4 Department of Obstetrics & Gynaecology, Faculty of Clinical Sciences, College of Medicine, University of Lagos, Idi-Araba, Lagos, Nigeria; 5 Centre for Clinical Trials and Implementation Science (CCTRIS), College of Medicine, University of Lagos, Idi-Araba, Lagos, Nigeria; 6 Maternal, Adolescent, Reproductive and Child Health (MARCH), Centre, London School of Hygiene and Tropical Medicine, London, United Kingdom; 7 Department of Medicine, Faculty of Clinical Sciences, College of Medicine, University of Lagos/Lagos University Teaching Hospital, Idi-Araba, Lagos, Nigeria; 8 International Research Center of Excellence, Institute of Human Virology Nigeria, Abuja, Nigeria; 9 Global Pediatrics Program and Division of Infectious Diseases, Department of Pediatrics, University of Minnesota Medical School, Minneapolis, Minnesota, United State of America; Emory University School of Medicine, UNITED STATES OF AMERICA

## Abstract

**Background:**

Anaemia in pregnancy (AIP), defined as a haemoglobin concentration of less than 11g/dl, is a significant global health issue, especially in resource-constrained settings. Of all the causes of AIP, iron deficiency anaemia is the commonest, accounting for approximately half of cases. While oral iron treatment is standard according to the WHO recommendation, poor adherence is a concern. Intravenous (IV) iron is an alternative treatment, especially in cases where oral iron is challenging to utilise. However, this intervention is yet to be integrated into routine antenatal care. This study aimed to assess skilled health personnels' (SHPs) perceptions of the acceptability and feasibility of IV iron in managing AIP in Lagos and Kano based on SHP characteristics and changes over time.

**Methods:**

This was a repeated cross-sectional study embedded within a randomised controlled trial (RCT) conducted in 11 healthcare facilities in Lagos and Kano states, Nigeria. All SHPs trained in implementing IV iron were invited to participate in the study. Data were collected using the Acceptability and Feasibility of Intervention measure (AIM and FIM) survey tools between the RCT baseline in August 2021 and the endline in May 2023. Analysis was done using descriptive statistics, independent and Paired T-tests and Analysis of variance (ANOVA) in Stata.

**Results:**

Of the 60 SHPs invited at baseline (pre-implementation), 53 (88%) completed the survey. At endline (post-implementation), 39 of the 46 SHPs involved in IV iron implementation responded (85%). When comparing groups, baseline AIM scores differed significantly by state (*p* = 0.001), though this was not sustained at endline. No other significant differences were seen by cadre or facility for AIM, and FIM did not differ significantly by state, cadre, or facility at either time point. Among the 20 SHPs who completed both surveys, the AIM score increased from baseline 16.8/20 (Standard deviation, SD = 2.0) to endline (18.5, SD = 2.0) (*p* = 0.002). FIM scores also increased over time, 17.1/20 (SD = 2.3) versus 18.2/20 (SD = 1.7) (*p* = 0.088).

**Conclusion:**

Post-implementation, IV iron was perceived as highly acceptable and feasible with no differences across cadre, state, or facility levels. While acceptability significantly improved over time, feasibility also increased, though not statistically significantly at the 0.05 level.

## Introduction

Anaemia in pregnancy (AIP) is a highly prevalent condition of global health concern [1,2]. AIP occurs when the haemoglobin (Hb) concentration is less than 11g/dl in the first and third trimester and less than 10.5g/dl in the second trimester [3]. This condition poses significant risks to women and their babies, especially in low and middle-income countries (LMICs) like Nigeria [2,4,5]. This risk increases when AIP is left untreated, negatively impacting perinatal outcomes from complications such as intrauterine growth restriction, stillbirth, premature delivery, low birth weight, neonatal death, amongst others [1,6]. Additionally, AIP poses risks for poor maternal outcomes, including maternal mortality, particularly in women suffering from severe anaemia [6,7]. Studies have shown that iron deficiency anaemia (IDA) is the leading cause of AIP [2,8]. While IDA can negatively impact the mother, it also poses risks to the foetus, potentially leading to neonatal neurocognitive impairment, including mental health challenges, amongst others [9,10]. Emerging neuroimaging evidence also suggests that in utero exposure to anaemia may affect foetal brain development involved in cognitive functions, increasing the risk of long-term cognitive difficulties in childhood [11]. The World Health Organization (WHO) recommends using iron supplements for the prevention and treatment of IDA and to mitigate its effects during pregnancy [12]. While oral iron supplements are readily available at low cost, they are often associated with poor tolerance and suboptimal adherence by pregnant women [10,13–16].

To address these issues associated with oral iron, some studies have demonstrated that intravenous iron (IV), is a safe and effective alternative therapy for moderate to severe IDA during pregnancy [17–20]. A systematic review and meta-analysis by Govindappagari et al. showed that IV iron therapy is two to three times more likely to rapidly correct IDA, with desired haemoglobin achieved within four weeks of treatment therefore, prompt intervention is crucial for good outcome [21]. This intervention not only proves to be clinically effective but also has the potential to significantly reduce overall treatment costs for users [22]. Additionally, it may alleviate the financial burden on health systems by minimising expenses related to managing complications associated with IDA [22].

However, despite its considerable potential, the use of IV iron poses challenges in LMICs, including Nigeria [16,23]. These challenges are multifaceted and can contribute to its underutilisation [16,23]. For instance, from the users’ perspective, lack of awareness and fear of needles can serve as deterrent from utilising this intervention [16,23]. In contrast, from the perspective of skilled health personnel (SHPs) tasked with administering IV iron, preconceived notions about adverse events associated with IV iron, lack of training, and a limited number of providers can further hinder its utilisation [16,23]. Therefore, it is important to carefully assess the acceptability and feasibility of IV iron in the Nigerian healthcare settings where these challenges exist, to develop mitigation strategies supporting its use.

Acceptability of an intervention refers to stakeholders’ perception that a treatment is agreeable, while feasibility relates to how the new treatment can be successfully implemented within a setting [24,25]. Both acceptability and feasibility are essential implementation outcomes, especially in the context of the Nigerian healthcare system, which has substantially fewer SHPs than WHO recommends [26]. Previous studies highlighted that a shortage of personnel could hinder the acceptability and safe administration of IV iron [16,27]. Already busy schedules of the limited SHPs, coupled with the extra effort required for the safe administration of IV iron (administered within 15–20 minutes, with 30 minutes of close observation post-administration) could increase workload [16,27–29]. Such burden negatively affects plans for routinising IV iron into antenatal care (ANC) practice and hence its feasibility, unless the acceptability and feasibility of its utilisation are determined [16,27,29].

While evidence in the literature highlights potential factors that can influence the acceptability of IV iron among SHPs [16,27], there is a lack of understanding of their perceptions of this intervention after implementation. Additionally, there is a gap in the patterns and trends in the perceptions of SHPs of the acceptability and feasibility of integrating IV iron into routine ANC practice over time as SHPs are exposed to and gain experience using IV iron. Gaining insights into the acceptability and feasibility of integrating IV iron treatment of AIP within healthcare facilities is crucial for successful implementation and generating recommendations for scaling up and sustainability [30,31]. Our objective in this study was to assess SHP perception of the acceptability of IV iron and the feasibility of its integration into the existing workflow of treating AIP in Lagos and Kano states, Nigeria, examining patterns based on SHP characteristics and changes over time.

## Methods

### Study design

This was a repeated cross-sectional study conducted at two time points (baseline and endline of a trial) in healthcare facilities where women with AIP were treated using IV iron therapy. This study was embedded within the context of an open-label Intravenous versus Oral Iron for Anaemia in Pregnant Nigerian Women (IVON) trial with registration numbers ISRCTN63484804 and NCT04976179 respectively [32]. This trial was a randomised controlled trial and implementation study based on a hybrid study design [32]. We utilised the Strengthening the Reporting of Observational Studies in Epidemiology (STROBE) checklist to report our findings [33].

### Study setting

This study was conducted in the two most populous states in Nigeria – Lagos and Kano [34,35]. Lagos is situated in the south-western region, and Kano is in the north-western region of the country [3435]. We selected these states due to the differing prevalences of moderate to severe AIP; the prevalence of moderate to severe AIP in the southwestern region was lower (22.5%), compared to the northwestern region at 32.5% [36]. Specifically, we conducted this study in all 11 public health facilities involved in the IVON trial, including two primary health centres (PHCs) in Lagos, three PHCs in Kano, and two secondary and one tertiary facility in each state [32].

### Participant sampling and recruitment

All SHPs trained to administer IV iron (baseline) and who had administered at least one dose of IV iron during the IVON trial (endline) were selected and invited to participate in the survey. The IV iron formulation administered during the IVON trial was Ferric Carboxymaltose (FCM). This dextran-free, IV iron formulation, has a near-neutral potential of hydrogen (pH) that has been proven to be safe and effective for treating IDA in the second and third trimesters of pregnancy [14]. We utilised the total population sampling technique to ensure responses from all trained SHPs with experience administering FCM in all facilities involved in the IVON trial. SHPs who were not trained, had not administered FCM or declined consent were excluded from the survey.

Following training on safe administration of FCM at baseline of the IVON trial (pre-implementation phase), all SHPs were informed about the study. Those who gave verbal consent to participate in the study were asked for their email addresses and sent an email with a unique link to a structured online survey on Research Electronic Data Capture (REDCap) (Vanderbilt University, Nashville, Tennessee, US). The survey communicated the study objectives and the voluntary nature of participation. SHPs who provided written informed consent before starting the online survey received a prompt to proceed with the survey with complete responses. At the end of the IVON trial (endline), the same survey was repeated and sent via email to the addresses of consented SHPs who administered at least one FCM in the trial.

### Study variables

This study included both outcome and independent variables centred on the SHPs’ perceptions of the acceptability and feasibility of IV iron, examining changes over time and differences across professional and contextual groups.

### Outcome variables

The acceptability and feasibility of FCM administration within the healthcare facilities were the outcome variables. We defined acceptability as SHPs’ perception that FCM was agreeable or satisfactory [24,25]. Feasibility was the degree to which FCM could be successfully utilised within the healthcare facilities [24,25].

### Independent variables

The primary independent variables were treated as between-group factors and included key group-level characteristics: cadre (doctor vs. nurse/nurse midwife), state (Lagos vs. Kano), and facility level (primary, secondary, tertiary). These variables were used to examine cross-sectional differences in perceived acceptability and feasibility of IV iron at each time point. The secondary independent variable was time point, treated as a within-group factor with two levels (baseline and endline), representing the period before and after the implementation of IV iron.

### Data collection

The Acceptability of Intervention Measure (AIM) and the Feasibility of Intervention Measure (FIM) tools were used to measure the outcomes of interest [31]. The AIM and FIM tools have demonstrated acceptable content validity, structural validity, known-group validity and test-to-retest reliability [31]. In the Nigerian context, the internal consistency of the scales was satisfactory, with Cronbach’s alpha values of 0.85 for AIM and 0.75 for FIM. Each tool comprised four questions centred around the acceptability and the feasibility of utilising FCM to treat AIP [S1 and S2 Files]. The AIM survey tool included questions such as “FCM for AIP meets my approval,” “FCM for AIP is appealing to me,” “I like FCM for AIP,” and “I welcome FCM for AIP.” The FIM survey tool similarly had questions like, “FCM for AIP seems implementable,” “FCM for AIP seems possible,” “FCM for AIP seems doable,” and “FCM for AIP seems easy to use.” Each question was scored on a 5-point Likert scale ranging from 1 to 5 (1 = completely disagree, 2 = disagree, 3 = neither agree nor disagree, 4 = agree, 5 = completely agree). The total scores ranged from 5 to 20 for each measure, with 5 being the lowest and 20 being the highest. Considering the absence of a standardised cut-off, we defined ad hoc levels of acceptability and feasibility as follows: a low score is 5–9, a moderate score is 10–14, and a high score is 15–20.

In addition to the above questions, the survey also inquired about the characteristics of the SHP, such as age group (18–24, 25–39, 40–49, 50–59 years), gender (male, female), cadre (doctor, nurse/nurse midwives), duration of clinical service, health facility (primary, secondary, tertiary), state (Lagos, Kano) etc. [S1 and S2 File]. SHP cadre, facility level and state were selected as independent variables in this study

All data were collected using electronic devices between the 1st and 8^th^ of August 2021 (baseline) or from the 1^st^ of May to the 15^th^ of June 2023 (endline). To ensure that each survey is completed, the software prompts the participant to respond to all questions before advancing to the next section. As a result, there was no item-level missingness.

### Protocol for FCM administration

During the IVON trial, FCM was given as a single dose of 20 mg/kg of body weight, with a maximum dose of 1000 mg, to pregnant women with a Hb concentration of less than 10g/dl who had no contraindications for use [19,37]. Contraindications included previous reactions to any IV iron formulation, severe respiratory or cardiac disease, or a history of other drug allergies [38,39]. SHPs administered FCM in the outpatient ANC setting across the 11 participating health facilities (ranging from primary to tertiary facilities) to eligible pregnant women who provided consent and were randomised into the intervention arm of the trial.

Before the administration of FCM, the responsible SHP ensured emergency readiness by ascertaining the availability of resuscitation drugs (such as adrenaline, hydrocortisone, etc) and oxygen. Baseline vital signs were then observed. Following the establishment of IV access, FCM was dispensed into 200 ml of normal saline infusion at the individual dose in kg per body weight. Each infusion was initially administered at a low flow rate to assess early-onset adverse events, after which vital signs were monitored in the first two minutes. If there was no reaction to the FCM, the flow rate was increased for completion in 15–20 minutes. Subsequently, an immediate and 30-minute post-administration vital signs check was done before the patient was discharged [32].

### Implementation strategies

Prior to commencing the trial, contextual factors with the potential to influence the implementation of FCM were explored [16]. These factors enabled the development of targeted strategies to improve the implementation of FCM, with the aim of achieving the outcome measures (acceptability and feasibility of FCM) [27]. All SHPs, including skilled research nurses (employed by the trial), facility-based nurses/midwives who assisted the research nurses as needed, and medical doctors specialising in feto-maternal care involved in the IVON trial in each healthcare facility received training before the trial (pre-implementation) to mitigate risks of adverse events associated with FCM administration. The research team provided a treatment protocol/standard operating procedure (SOP) and a step-by-step guide on FCM administration. The research team also made emergency drugs/materials and equipment available for prompt response in case of adverse reactions during FCM administration. During the trial (implementation phase), the SHPs underwent six-monthly refresher training on FCM administration. Furthermore, the research team monitored progress and consistently engaged with the SHPs to enhance the quality of care, fostering a culture of collaborative teamwork among them [Table 1].

**Table 1:** Implementation strategies identified for the successful implementation of FCM.

Phase	Implementation strategies	Implementation outcomes of interest
Pre-implementation phase	Engaged skilled research SHPs	Acceptability Feasibility
	SHP training on basic life support	
	Baseline SHP training on the safe administration of FCM	
	Provision of treatment protocol, guidelines, counselling charts and algorithm	
	Provision of resuscitative drugs/ materials and equipment	
Implementation phase	Periodic SHP training on the safe administration of FCM	
	Regular monitoring of progress and continuous engagement to improve the quality of care	
	Collaborative teams between research and facility SHPs	

*SHP, Skilled Health Personnel*

### Data analysis

We conducted a descriptive analysis of the characteristics of SHPs who responded to the surveys and presented the results as categorical variables using frequencies and percentages and continuous variables using means and standard deviations (SD). We calculated the frequency and percentage distribution of SHP responses to each item on the AIM and FIM surveys. Composite scores for each measure were obtained by adding the four individual questions of the survey tools, resulting in a total score for each intervention measure (AIM and FIM) at baseline and endline, without imputation. Analysis was performed using complete-case data. We calculated the mean and SD of the composite scores for both time points.

After confirming normality (Shapiro–Wilk) and homogeneity of variances (Levene), we compared differences in mean scores between AIM and FIM by cadre and state at baseline and endline respectively, using independent (two-sample) t-test. We utilised one-way ANOVA to examine differences in AIM and FIM scores by facility level at both time points. A post hoc Bonferroni test was conducted to identify the specific facility level with statistically significant differences. To account for multiple between-group comparisons (cadre, state and facility), a Bonferroni correction was applied, setting the significance level at p < 0.0083.

Furthermore, we performed a within-group analysis among the subset of SHPs who completed both the baseline and endline surveys. For this analysis, we used a paired t-test to ascertain the difference between AIM and FIM mean scores over time, comparing baseline and endline. Mean difference, SD, and 95% confidence interval were presented, and a p-value of less than 0.05 was considered statistically significant for the paired comparison. Effect sizes were reported for all comparisons (Cohen’s *dz* for paired tests, Cohen’s *d* for independent tests, and η² for ANOVA), with confidence intervals.

We conducted data analysis using Stata version 17.0 statistical software (StataCorp LLC, College Station, Texas, US).

### Ethics

Ethical approval was obtained from the National (NHREC/01/01/2007–17/01/2021), Lagos State (LSHSC/2222/VOLIII), Kano State (MOH/Off/797/T.1/2102), Health Research and Ethics Committees of the Lagos University Teaching Hospital (ADM/DCST/HREC/APP/3971), and Aminu Kano Teaching Hospital, Kano State (NHREC/28/01/2020/AKTH/EC/2955). Verbal consent was obtained to contact all SHPs, and written informed consent was obtained from all participants online before each survey.

## Results

**Fig 1:**
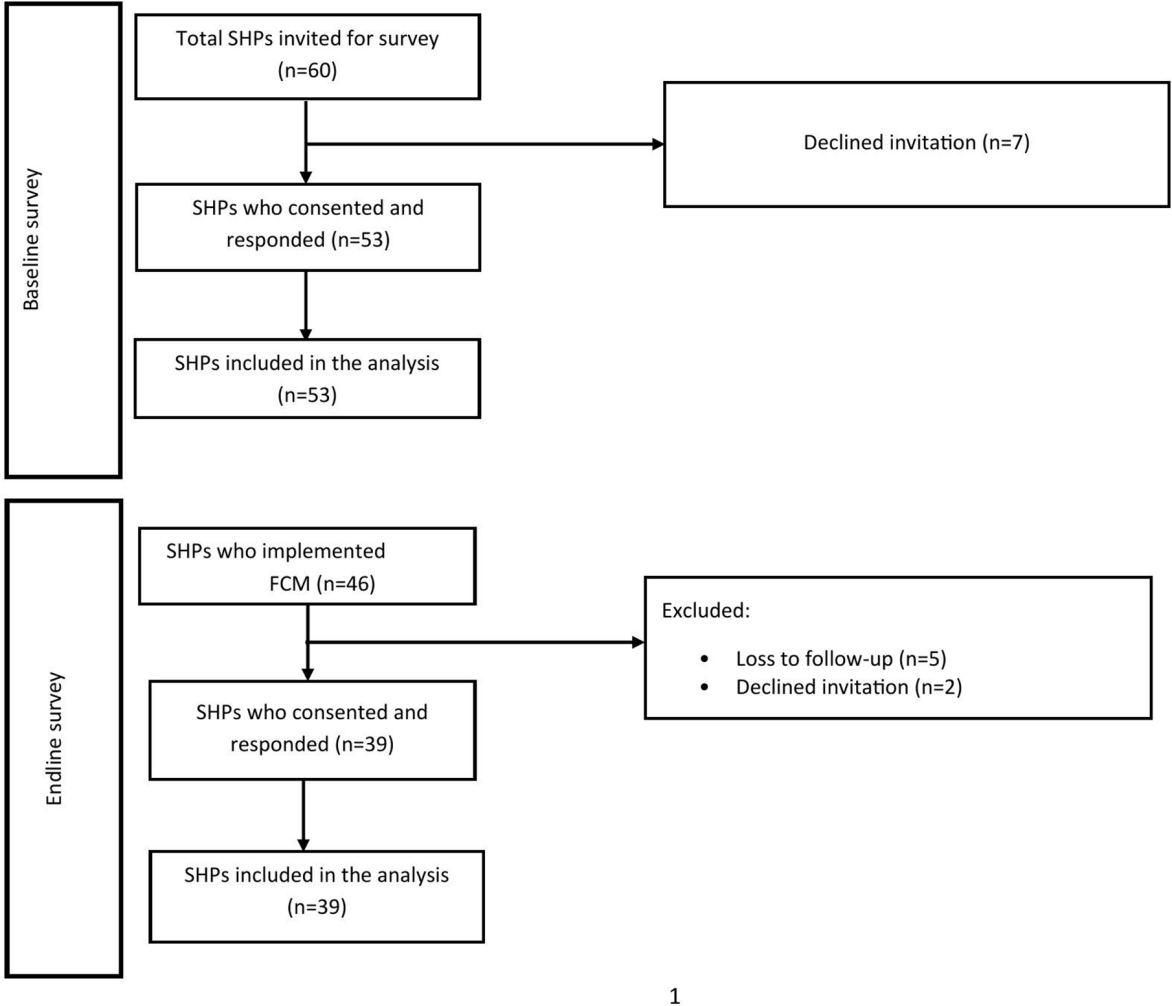
Number of SHPs at baseline and endline. SHPs, Skilled Health Personnel; FCM, Ferric Carboxymaltose.

In total, sixty SHPs (i.e., all trained SHPs) were invited to participate in the baseline survey, with 53 (88%) of them completing it. Following the training, 46 SHPs implemented IV iron, all of whom were invited to complete the endline survey. Of these 46 SHPs, 39 completed the survey at endline (85%). Of all those recruited, 20 SHPs participated in both the baseline and endline surveys [Fig 1].

The SHPs surveyed were predominantly female; baseline 36/53 (68%) and endline 27/39 (69%). The participants were mainly medical doctors at both baseline, 29/53 (55%), and 21/39 endline (54%), mostly in the 25–39 years group, 30/53 (57%) at baseline, and 21/39 (54%) at endline. In line with the overall SHP distribution in the IVON trial, most SHPs were from secondary healthcare facilities, with 26/53 (49%) at baseline, and 23/39 (59%) at endline. The mean years of clinical practice experience were 12.0 (SD = 8.88) at baseline and 9.0 (SD = 6.65) at endline [Table 2].

**Table 2:** Characteristics of survey respondents (SHP) at baseline and endline.

Characteristics of participants	Category	Baseline	Endline
		Frequency (n = 53)	Frequency (n = 39)
**Gender**	Male	17 (32.1)	12 (30.8)
	Female	36 (67.9)	27 (69.2)
**SHP cadre**	Doctor	29 (54.7)	21 (53.8)
	Nurse/ nurse-midwife	24 (45.3)	18 (46.2)
**Age group**	18 −24 years	0 (0.0)	3 (7.7)
	25 −39 years	30 (56.6)	21 (53.8)
	40-49 years	13 (24.5)	9 (23.1)
	50-59 years	10 (18.9)	6 (15.4)
**Facility level**	Primary	10 (18.8)	8 (20.5)
	Secondary	26 (49.1)	23 (59.0)
	Tertiary	17 (32.1)	8 (20.5)
**State**	Lagos	33 (62.3)	22 (56.4)
	Kano	20 (37.7)	17 (43.6)

### Distribution of FCM administration across facilities

SHPs trained during the IVON trial delivered FCM to all pregnant women assigned to the IV iron arm (n = 527) across the 11 participating healthcare facilities. Most administrations occurred in secondary facilities (n = 359, 68.1%), followed by primary facilities (n = 126, 23.9%), with fewer in tertiary facilities (n = 42, 8.0%).

### Perception of SHP of acceptability and feasibility

**Fig 2:**
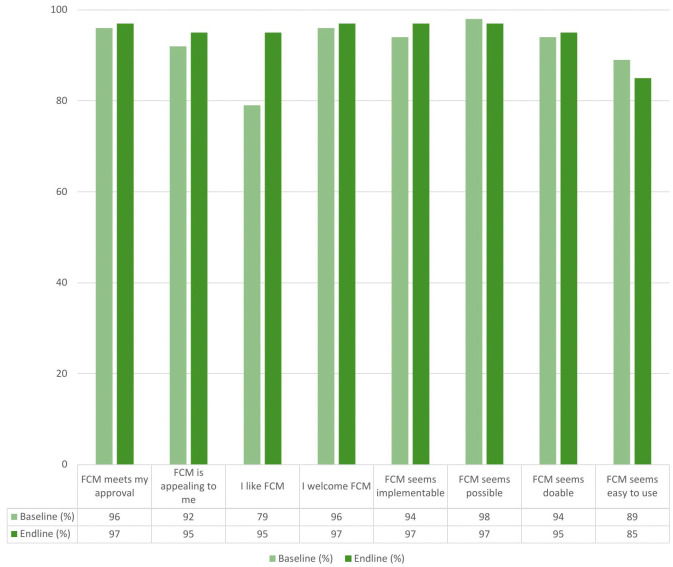
Acceptability and feasibility of FCM utilisation for IDA treatment among skilled health personnel. FCM, Ferric Carboxymaltose; IDA, Iron Deficiency Anaemia.

The majority of the respondents, 51/53 (96%) at baseline and 38/39 (97%) at endline, agreed/completely agreed that FCM met their approval, while 49/53 (92%) at baseline and 37/39 (95%) at endline agreed/completely agreed that it was appealing. Additionally, most respondents, 42/53 (79%) at baseline and 37/39 (95%) at endline, agreed/completely agreed that they liked FCM. Almost all participants, 51/53 (96%) at baseline and 38/39 (97%) at endline, agreed/completely agreed that they welcomed the use of FCM [Fig 2].

Regarding the feasibility of utilising FCM for treating AIP, the majority of the SHPs agreed/ completely agreed that FCM seems implementable at the baseline (50/53, 94%), and at the endline (38/39, 97%). Additionally, almost all the SHPs agreed/completely agreed that FCM seems possible at the baseline (52/53, 98%) and endline (38/39, 97%) and doable at the baseline (50/53, 94%) and endline (37/39, 95%). Regarding ease of use, the majority of participants found FCM easy to use at baseline (47/53, 89%) and endline (33/39, 85%) [Fig 2].

### Comparison of the outcome measures scores by SHP cadre, state, facility level and timepoints

At baseline, the mean AIM total score (n = 53) was 17.1 (SD = 2.1). At the endline (n = 39), the mean score was 18.3 (SD = 1.9). Notably, nurse/nurse-midwife consistently achieved higher scores at both the baseline (17.3, SD = 2.0) and endline (18.8, SD = 1.3) than doctors. However, there was no statistically significant difference at either the baseline or endline (*p* > 0.05). The corresponding effect size was Cohen’s d = –0.49 (95% CI: –1.13 to 0.15) at baseline and –0.24 (95% CI: –0.78 to 0.30) at endline.

When analysing the mean AIM score by state, SHPs from Kano consistently scored higher than those from Lagos at both the baseline (18.2, SD = 1.7) and endline (18.6, SD = 1.8) assessments. At the baseline, the difference in mean score was statistically significant (*p* = 0.001). The effect size for the between-state difference at baseline was Cohen’s d = –0.29 (95% CI: –0.92 to 0.35), and at endline, it was Cohen’s d = –0.96 (95% CI: –1.54 to –0.37).

At the facility level, SHPs working in PHC facilities had a higher mean score (17.4, SD = 1.9) than those in secondary (17.0, SD = 1.9) and tertiary (17.0, SD = 2.6) facilities. However, at the endline, tertiary facilities had a higher mean AIM score (19.2, SD = 1.4) than the other facility types. Nonetheless, there were no statistically significant differences between the groups at either baseline or endline (*p* > 0.05). The corresponding effect size at baseline was η² = 0.01 (95% CI 0.00 to 0.06), and at endline, it was η² = 0.06 (95% CI 0.00 to 0.22). [Table 3].

**Table 3:** The comparison of the AIM and FIM scores by cadre, state and facility level.

AIM score					FIM score			
Categories	Mean (SD)		Mean difference (95% CI)	p-value	Mean (SD)		Mean difference (95% CI)	*p-*value
	Total mean score				Total mean score			
Baseline (n = 53)	17.1 (2.11) ^a^		**–**	**–**	17.0 (2.19) ^a^		**–**	**–**
Endline (n = 39)	18.3 (1.92) ^a^		**–**	**–**	17.4 (2.11) ^a^		**–**	**–**
**Group differences**								
**SHP Cadre**	**Doctor**	**Nurse/nurse-midwife**			**Doctor**	**Nurse/nurse-midwife**		
Baseline (n = 53)	16.8 (2.1)	17.3 (2.0)	0.5 (−0.6–1.6)	0.385 ^a^	17.2 (2.4)	16.8 (2.0)	−0.4 (−1.6–0.8)	0.498 ^a^
Endline (n = 39)	17.9 (2.2)	18.8 (1.3)	0.9 (−0.3 - 2.1)	0.135 ^a^	17.3 (2.0)	17.5 (2.2)	0.1 (−1.2–1.5)	0.801 ^a^
**State**	**Lagos**	**Kano**			**Lagos**	**Kano**		
Baseline (n = 53)	16.3 (2.0)	18.2 (1.7)	1.8 (0.7–2.9)	**0.001** ^**a**^	17.1 (2.3)	17.0 (2.0)	−0.2 (−1.4–1.0)	0.762 ^a^
Endline (n = 39)	18.0 (2.0)	18.6 (1.8)	0.5 (−0.7–1.8)	0.378 ^a^	17.5 (2.0)	17.4 (2.2)	−0.1 (−1.4–1.3)	0.900 ^a^
	**Mean (SD)**			**F-statistics (p-value)**	**Mean (SD)**			**F-statistics (*p-*value)**
**Facility level**	**Primary**	**Secondary**	**Tertiary**	**–**	**Primary**	**Secondary**	**Tertiary**	**–**
Baseline (n = 53)	17.4 (1.9)	17.0 (1.9)	17.0 (2.6)	0.13 (0.880) ^b^	16.9 (2.6)	16.9 (2.1)	17.1 (2.1)	0.60 (0.93) ^b^
Endline (n = 39)	18.2 (2.3)	18.0 (1.9)	19.2 (1.4)	1.20 (0.317) ^b^	17.2 (2.6)	17.3 (2.0)	18.1 (2.2)	0.48 (0.620) ^b^

AIM, Acceptability of Intervention Measures; FIM, Feasibility of Intervention Measures; SD, standard deviation; CI, confidence interval. Hypothesis testing done with a. independent t-test (between-group), and b.one-way ANOVA (between groups, i.e., 3-group comparison). Subgroup sample sizes at each time point are reported in Table 2.

The mean FIM score at baseline (n = 53) was 17.0 (SD = 2.1), while at the endline (n = 39), the mean score was 17.4 (SD = 2.1). At baseline, doctors had a higher mean score of 17.2 (SD = 2.4) than nurse/nurse-midwife, who had a mean score of 16.8 (SD = 2.0). However, at the endline, nurse/nurse-midwife had a higher mean score of 17.5 (SD = 2.2) than doctors, who had a mean score of 17.3 (SD = 2.0). The differences between the groups at both time points were not significant (*p* > 0.05). For FIM, the effect size for the doctor versus nurse/nurse-midwives comparison at baseline was Cohen’s d = –0.08 (95% CI: –0.71 to 0.55), and at endline Cohen’s d = 0.19 (95% CI: –0.35 to 0.73).

In Lagos, the SHPs had higher mean FIM scores than those in Kano state at both baseline (17.1, SD = 2.3) and endline (17.5, SD = 2.0). However, the differences were not statistically significant (p > 0.05). The effect size for the between-state difference at baseline was Cohen’s d = 0.04 (95% CI: –0.59 to 0.67). At endline, the corresponding effect size was Cohen’s d = –0.96 (95% CI: –1.54 to –0.37).

At the facility level, SHPs in tertiary healthcare facilities consistently scored higher at both baseline (17.1, SD = 2.1) and endline (18.1, SD = 2.2). Similarly, these differences were also not statistically significant (*p* > 0.05). The effect size at baseline was η² = 0.003 (95% CI: 0.00 to 0.03), while at endline it was η² = 0.03 (95% CI: 0.00 to 0.15). [Table 3].

Among the subset of SHPs who participated in both the baseline and endline surveys (n = 20), the mean AIM and FIM score increased over time. Specifically, at baseline, the mean AIM score was 16.8 (SD = 2.0), which increased to 18.5 (SD = 2.0) at endline. This change in the AIM score was statistically significant (*p* = 0.002), with a mean difference of 1.6 (95% CI: 0.6–2.6) and an effect size of Cohen’s dz = 0.78 (95% CI: 0.34–1.22).

**Fig 3:**
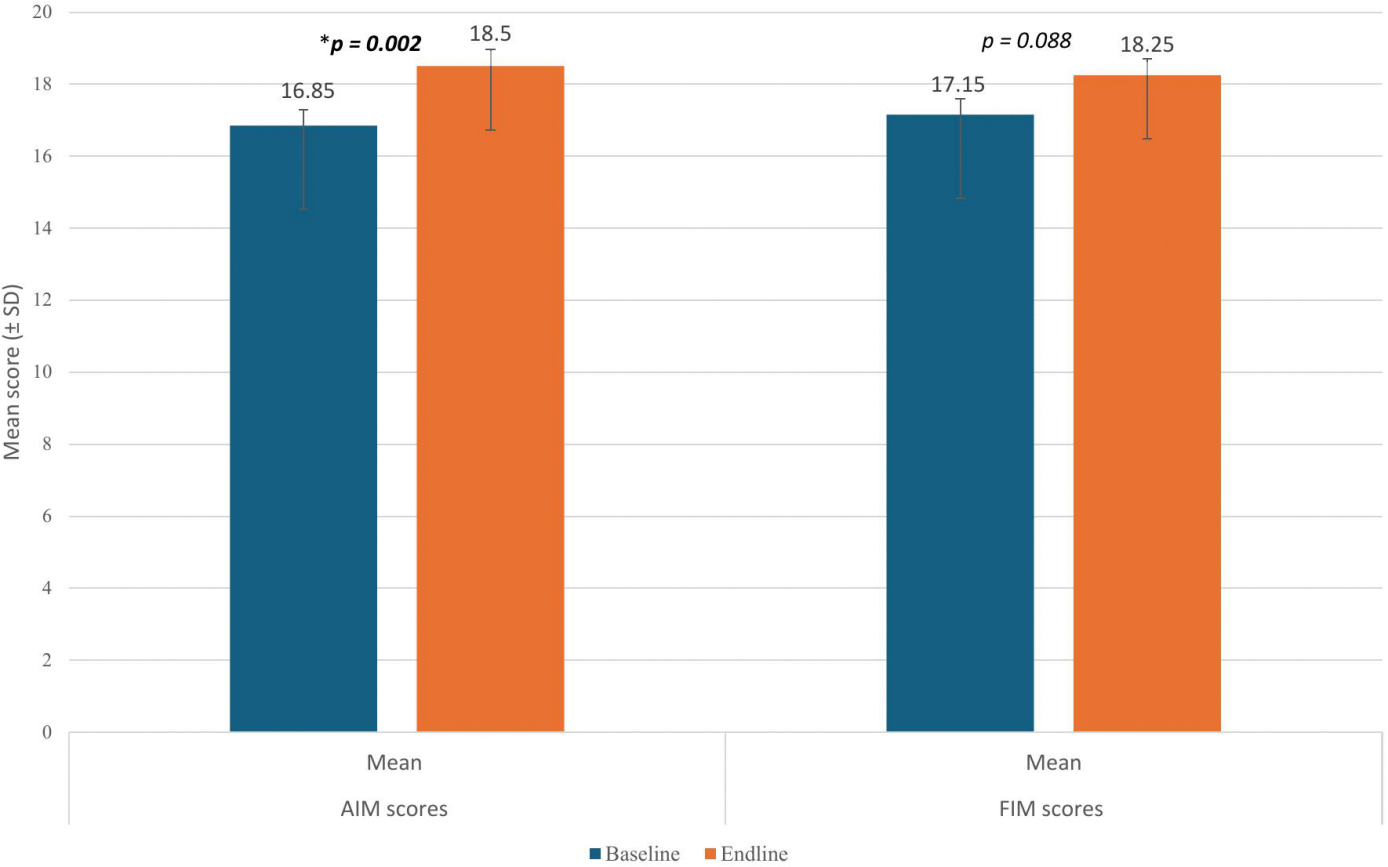
Comparison of mean AIM and FIM scores at baseline and endline among matched SHPs (n = 20). AIM, Acceptability of Intervention Measures; FIM, Feasibility of Intervention Measures; SD, Standard Deviation; CI, Confidence Interval. AIM mean difference: 1.6 (95% CI: 0.6–2.6). FIM mean difference: 1.1 (95% CI: −0.1–2.3). Error bars show SD.

In contrast, the mean FIM score also improved, increasing from 17.1 (SD = 2.3) at baseline to 18.2 (SD = 1.7) at endline with a mean difference of 1.1 (95% CI: −0.1–2.3); however, this change was not statistically significant (*p* = 0.088), and the effect size was Cohen’s dz = 0.40 (95% CI: 0.02–0.78) [Fig 3].

## Discussion

This repeated cross-sectional study assessed the perception of the acceptability of IV iron and the feasibility of integrating it into the existing workflow for treating AIP in two states in Nigeria, focusing on differences by SHPs’ characteristics and changes over time. The study found that the perceived acceptability and feasibility of integrating IV iron into daily practice were high, as indicated by total AIM mean scores of 17.1 at baseline and 18.3 at the endline, as well as a total FIM mean scores of 17.0 and 17.4 at both time points. The study also found significant differences in mean AIM scores by practice location (Kano state) at the baseline (p = 0.001) and by time point (p = 0.002).

The high perceived acceptability of IV iron at both time points reflects the positive perception of the SHPs towards it as an intervention to treat AIP in healthcare facilities. This finding is consistent with a previous study and is critical for intervention acceptability [29]. The observed high acceptance rate can be attributed to targeted strategies designed to seamlessly integrate this intervention within the context of a closely monitored RCT [16]. These strategies were specifically tailored to address the needs of SHPs, providing them with the necessary training, equipment, supplies and monitoring to administer IV iron safely and confidently [25].

Across both the baseline and endline surveys, nurses had higher AIM scores than doctors. However, the differences were not statistically significant (*p* > 0.05). This observation suggests that there is no difference in perception by the cadre. This consistent acceptability implies that IV iron was regarded as both relevant and practical, irrespective of the varied roles involved in its administration. Given that both doctors and nurses often share the responsibilities of implementing clinical interventions, their aligned perceptions facilitate a more seamless integration of IV iron therapy into everyday clinical workflows. For instance, while the nurses had a more focused role in the administration of IV iron during the RCT, the doctors remained on-site to monitor the administration and respond promptly to any potential adverse events [38,40,41]. This inter-cadre agreement, which was enhanced through effective implementation strategies like training, is crucial for patient safety and intervention effectiveness [25]. It is important to note that this implementation strategy not only allayed the SHPs’ concerns but also equipped them with the necessary skills and knowledge, ultimately enhancing the acceptability of IV iron as an intervention [16,27].

In addition, our study demonstrated a statistically significant difference in the mean AIM score, with Kano having a higher score than Lagos State at baseline (mean difference = 1.8, *p* = 0.001). However, this difference was not sustained at endline (*p* = 0.378), suggesting convergence over time between the two states. This initial significant difference may have stemmed from effectively utilising freely available IV iron during the trial to treat the highly prevalent moderate to severe anaemia in Kano state [20]. For instance, during the RCT, it was observed that 51% of pregnant women in Kano State had AIP, surpassing the rates in Lagos State (49%) [20]. This high prevalence can be traced to various contextual factors: women in northwestern geopolitical zones where Kano State is situated tend to have lower socioeconomic status, with limited access to education and healthcare resources, all of which are well-documented risk factors for IDA and increase their risk of AIP [2,42]. The free access to IV iron throughout the RCT may have played a crucial role in fostering higher acceptance of this intervention among SHPs in Kano, as they witnessed its direct impact on addressing the high prevalence of anaemia in their setting [20]. However, the attenuation of this difference by the endline suggests that, once the SHPs experienced the benefits of IV iron, there was greater consistency in their acceptance, reflecting a broader understanding and alignment with its use as a treatment option.

No significant differences were observed at either time point by facility type (*p* > 0.05). This observation suggests that SHPs across all tiers of the healthcare system perceived the intervention in a remarkably similar way, regardless of the structural differences and care-delivery models at different levels. At both the baseline and endline, it is evident that the majority of the SHPs view IV iron as implementable, possible, doable and easy to use and like acceptability, we found no significant differences across characteristics, including cadre, state, and facility type (p > 0.05).

Further investigation into the subset of SHPs who participated in both the baseline and endline surveys (n = 20) showed that the mean scores for the AIM and FIM measures increased over time. However, the significance of these changes varied between the two measures. For instance, the mean AIM score increased significantly, rising from 16.8 at baseline to 18.5 at endline (p = 0.002), corresponding to a mean difference of 1.6 on a 20-point scale. This observation indicates that the SHPs viewed the intervention more positively with continued exposure, reflecting their growing confidence in its implementation. Conversely, while the mean FIM score also increased, from 17.1 to 18.2, this change was not statistically significant (p > 0.05). This modest improvement, with a mean difference of 1.1, suggests that while IV iron has become more acceptable among SHPs, there may still be lingering concerns regarding practical or system-level barriers that hinder its integration into routine practice. Although no established threshold currently exists for interpreting the clinical relevance of changes on the AIM or FIM scales, our findings highlight the difference between an intervention meeting the needs of SHPs and its ability to fit seamlessly into existing workflows [25].

For instance, during the RCT, IV iron administration was scheduled during routine ANC outpatient visits, which might have conflicted with other routine clinical responsibilities, making it challenging for SHPs to incorporate the intervention into their daily practice [27,43]. Additionally, the shortage of SHPs exacerbated by the ongoing “Japa syndrome,” a wave of emigration to more developed countries, has left Nigeria with only 24,000 licensed medical officers, well below 10% of the WHO’s recommended minimum [26,44]. This workforce gap has resulted in a heavy workload and strain on remaining staff, which likely influenced SHPs’ perceptions of the feasibility of integrating IV iron into routine care during the study period [27,44]. In this context, even a well-accepted intervention can be challenging to integrate into existing workflows if it adds complexity or time pressure to routine tasks.

### Implication for practice, research and policy

Our study highlights the high acceptability and feasibility of integrating IV iron for treating AIP into routine antenatal care in Nigeria. While the strategies employed in this study appear to have improved the acceptability and, to some extent, the perceived feasibility of IV iron during the implementation period, it remains uncertain whether these effects will have lasting effects beyond the confines of the RCT. If their impact is not sustained over time, it could influence the SHPs’ perceptions of the intervention’s acceptability and feasibility. Therefore, to create a lasting impact beyond the RCT and into real-world settings, these strategies can be identified, tested and refined through implementation research, laying the foundation for future scale-up [45].

Addressing the concerns that SHP have regarding feasibility is essential before integration. Inadequate human resources remain a key barrier to integrating IV iron into routine care. While we involved skilled research nurses who closely collaborated with the facility SHPs to minimise the additional workload of administering IV iron in this study, such support is unlikely to be available in real-world practice. As a result, administering IV iron, particularly in busy ANC outpatient settings as done in this study, may be difficult to sustain without addressing workforce capacity [27,45,46]. Ensuring feasibility from the SHPs’ perspective will require practical adjustments to workflow and staffing in real-world implementation [16,27,45,46]. In the context of the continuous exodus of SHPs from the country, task shifting presents a feasible strategy to address critical workforce gaps in Nigeria’s healthcare system [16,27,45,46]. While task-shifting policies are already in place for certain cadres and services, we recommend expanding this approach to include IV iron administration [46,47]. To expand this policy safely and effectively, it will be essential to provide comprehensive training, clearly defined roles, and standardised competency assessments for the new cadres involved. Additionally, strong supervisory structures must be in place to ensure adherence to clinical protocols, enable prompt escalation of adverse events to SHPs, and safeguard the quality of care delivered to pregnant women [47].

Future research could enhance understanding by integrating the tools employed in this study (AIM and FIM surveys) with a qualitative approach in a mixed-methods study. This approach might provide a more comprehensive understanding of SHPs’ perceptions s, allowing for richer insights into their experiences and perspectives. In parallel, policy-focused research is equally important. Even though effective strategies are essential for integrating IV iron into routine care, its feasibility must be considered alongside the significant costs involved. For patients, financial burden may limit acceptance, while for the health system, costs extend beyond procurement to include sufficient human resources, SHPs’ training and developing comprehensive and standardised guidelines [16]. These realities highlight the urgent need for future research to evaluate the cost-effectiveness of IV iron therapy. Generating robust economic data is essential for informing policy and for strengthening provider confidence and system readiness for scale-up [48]. Addressing this current gap in future research is critical to ensuring that IV iron is not only clinically appropriate but also financially and operationally feasible for scale-up.

### Strengths and limitations

This study represents an implementation effort in Nigeria to assess the acceptability and feasibility of integrating IV iron administration into daily practice as implementation outcomes [49–51]. Specifically, we were able to comprehensively assess these outcomes by including the three levels of Nigeria’s healthcare system: primary facilities that offer basic antenatal services and limited staffing, secondary facilities that provide a wider range of services, and tertiary facilities that deliver specialised and advanced care. This multi-tiered approach allowed us to assess the distinct operational realities of each facility, strengthening the applicability of our findings across diverse healthcare contexts. Additionally, the AIM and FIM survey tools are structured and validated instruments that effectively measure implementation outcomes. Although concise, they have been widely used globally, ensuring reliable and applicable results in implementation science and providing valuable preliminary findings [31].

Although our study has several strengths, it is essential to acknowledge its limitations. For instance, following the formative evaluation conducted before the RCT, insights gathered from the SHPs influenced the decision to implement baseline training and refresher training at various intervals throughout the RCT period [16]. As a result, some responses obtained from the SHPs may have been shaped by the strategies put in place before collecting baseline data. This could have enhanced the acceptability and feasibility of IV iron among SHPs in this study, making it difficult to evaluate their perceptions prior to exposure to these strategies. Nevertheless, the difference observed between the baseline and endline responses, particularly regarding the perceived acceptability of IV iron, still supports the conclusion that these strategies are potent.

Furthermore, total-population sampling was employed, with all trained SHPs invited to participate in both surveys to capture the full range of perspectives. Although this approach was appropriate for the implementation context, the study was not powered to detect small subgroup differences. Additionally, the decline in the response rate from baseline to endline poses a potential risk of attrition bias, as non-respondents may have differing perceptions that could impact the observed trends. At endline, surveys were restricted to SHPs that had administered at least one dose of FCM, potentially introducing selection bias by excluding non-users and leading to higher acceptability and feasibility estimates. Moreover, there is a possibility of social desirability bias due to being self-reported. This bias was minimised by ensuring anonymity, as respondents completed the surveys privately and were informed that their responses would not be linked to individual SHPs. It is important to note that the observed patterns in this study may differ from those in other contexts, including private facilities.

Despite these limitations, our findings underscore areas for improvement and indicate the need for further research, particularly in developing strategies to enhance SHP engagement and ensure the long-term sustainability of IV iron therapy.

## Conclusion

The results of this study indicate that using IV iron as an intervention to treat AIP is not only highly acceptable but can also be integrated into routine antenatal practice, including in resource-limited settings like Nigeria. The high acceptability and feasibility can be attributed to targeted strategies implemented to enhance these outcomes within the context of a clinical trial. While there is a significant difference between the two time points in the acceptability of IV iron, there was no significant difference in the feasibility of integrating IV iron into routine practice in Nigeria. Hence, there is a need for further implementation research in real-world settings to evaluate these strategies and assess the practicability of utilising and integrating this intervention in routine practice, which could enhance scale-up and sustainability.

## Supporting information

S1 File Baseline AIMFIM survey tool. (DOCX)

S2 File Endline AIMFIM survey tool. (DOCX)

S3 File Additional data. (DOCX)

S4 File AIMFIM aggregate dataset. (XLSX)
