# Supplementary material for: Intravenous iron for anaemia in pregnancy: A quantitative study of acceptability and feasibility of its integration into routine antenatal care practice in Nigeria
Source: PLoS One. 2026 Feb 4;21(2):e0328239. doi: 10.1371/journal.pone.0328239 (PMC12871993; doi:10.1371/journal.pone.0328239)
Supplement: S2 File — (DOCX) [file pone.0328239.s002.docx]

**IVON Study: Acceptability of Intervention measure (AIM) and Feasibility of Intervention measure (FIM) Endline Assessment Tool**

This tool is to be administered among healthcare workers who have been trained and involved with the preparation, set-up, administration and patient/safety monitoring of IV iron at IVON study sites. It will take 15 to 20 minutes to complete.

**Consent Script:**

We have invited you for this survey as a healthcare provider who was trained and involved with the preparation, set-up, administration, and patient/safety monitoring of IV iron at IVON study sites on the acceptability and feasibility of implementing IV carboxymaltose as a new management strategy for anaemia in pregnancy. This online survey should take about 15 to 20 minutes to complete. Participation is voluntary, and responses will be kept confidential. You have the option to not respond to any questions that you choose. Participation or nonparticipation will not impact your relationship with the IVON-PP team. Submission of the survey will be interpreted as your informed consent to participate and that you affirm that you are at least 18 years of age.

If you have any questions about this survey, please contact:

**Principal Investigator: Prof. B.B Afolabi, email:** [**bbafolabi@unilag.edu.ng**](mailto:bbafolabi@unilag.edu.ng)

**OR: Co-investigator: Dr Nadia Sam-Agudu, email: nsamagudu@ihvnigeria.org**

**OR: Site-coordinator (LUTH): Dr Opeyemi Akinajo, opeyemiakinajo@gmail.com**

**Participants response: Kindly select any of the following option**

- I have read the above information and agree to participate in this research project. ____ (please tick)

- I have read the above information and do not agree to participate in this research project. ____ (please tick)

**Please print or save a copy of this page for your records.**

**Study Site Name:** **Study ID:** **AIM/FIM 01, 02 etc**.

**Section A: Participant Demographics**

1. Sex: □ Female □ Male
2. Age range: □18 to 24 yrs. □ 25 to 39 yrs. □ 40-49 yrs. □ 50-59 yrs. □ 60-69 yrs. □ 70 yrs.+
3. Health worker cadre: □ Nurse/nurse midwife □ Doctor □ Other (please specify)
4. Number of months/years in active clinical service (not training):
5. Hospital/clinic department, *e.g., Obstetrics and Gynaecology*:
6. Hospital/clinic unit, *e.g., labour ward or ANC clinic*:
7. Healthcare worker designation/title, *e.g., Chief Matron, Medical officer, Resident doctor, Consultant* etc.
8. What aspect of IV iron (ferric carboxymaltose) administration have you been involved with? Check all that apply:

**i. Pre-FCM administration phase:**

□ Ensuring availability of resuscitation medications and materials

□ Ensuring availability of study drug and infusion set inventory

□ Confirming patient’s randomization into the ferric carboxymaltose (FCM) group

□ Patient’s counselling and verbal consent-taking

□ Baseline vital signs check

□ Calculating and constituting appropriate FCM dose for administration

□ Preparing patients and securing IV access for administration

**ii. Intra FCM administration phase:**

□ Administering FCM

□ Mid-FCM administration vital signs check

iii. **Post FCM administration phase:**

□ Post FCM administration vital signs check

□ Observing the patient for at least 30 minutes

□ Documenting procedure in the patient’s case notes

1. Please indicate your responses by circling ONE answer for each question in the surveys below:

| **Section B: AIM Survey** |
| --- |

| **IVON Study Acceptability of Intervention Measure**  **IV=intravenous** | **Completely disagree** | **Disagree** | **Neither agree nor disagree** | **Agree** | **Completely agree** |
| --- | --- | --- | --- | --- | --- |
| **1. IV Iron for anaemia in pregnancy meets my approval.** | □ | □ | □ | □ | **□** |
| **2. IV Iron for anaemia in pregnancy is appealing to me.** | □ | □ | □ | □ | **□** |
| **3. I like IV Iron for anaemia in pregnancy.** | □ | □ | □ | □ | **□** |
| **4. I welcome IV Iron for anaemia in pregnancy.** | **□** | **□** | **□** | **□** | **□** |

| **Section C: FIM Survey** | | | | | | |
| --- | --- | --- | --- | --- | --- | --- |
| **IVON Study Feasibility of Intervention Measure**  **IV=intravenous** | **Completely disagree** | **Disagree** | **Neither agree nor disagree** | **Agree** | **Completely agree** |  |
| **1. IV Iron for anaemia in pregnancy seems implementable.** | □ | □ | □ | □ | □ |  |
| **2. IV Iron for anaemia in pregnancy seems possible.** | □ | □ | □ | □ | □ |  |
| **3. IV Iron for anaemia in pregnancy seems doable.** | □ | □ | □ | □ | □ |  |
| **4. IV Iron for anaemia in pregnancy seems easy to use.** | □ | □ | □ | □ | □ |  |

-------------------------------------------------------**Thank you for completing this survey**----------------------------

**Survey administrator name:**

**Date of survey completion:**
