# Supplementary material for: Intravenous iron for anaemia in pregnancy: A quantitative study of acceptability and feasibility of its integration into routine antenatal care practice in Nigeria
Source: PLoS One. 2026 Feb 4;21(2):e0328239. doi: 10.1371/journal.pone.0328239 (PMC12871993; doi:10.1371/journal.pone.0328239)
Supplement: S3 File — (DOCX) [file pone.0328239.s003.docx]

**Additional data not presented in the manuscript**

1. **Perception of SHPs on the acceptability of FCM**
2. **FCM for anaemia in pregnancy meets my approval**

**Baseline**

| **FCM for anaemia in pregnancy meets my approval** | **Frequency** | **Percent** | **Cum.** |
| --- | --- | --- | --- |
| Neither agree nor disagree | 1 | 5.00 | 5.00 |
| Agree and Completely agree | 19 | 95.00 | 100.00 |
| Total | 20 | 100.00 |  |

**Endline**

| **FCM for anaemia in pregnancy meets my approval** | **Frequency** | **Percent** | **Cum.** |
| --- | --- | --- | --- |
| Neither agree nor disagree | 1 | 5.00 | 5.00 |
| Agree and Completely agree | 19 | 95.00 | 100.00 |
| Total | 20 | 100.00 |  |

1. **FCM for anaemia in pregnancy is appealing to me**

**Baseline**

| **FCM for anaemia in pregnancy is appealing to me** | **Frequency** | **Percent** | **Cum.** |
| --- | --- | --- | --- |
| Neither agree nor disagree | 1 | 5.00 | 5.00 |
| Agree and Completely agree | 19 | 95.00 | 100.00 |
| Total | 20 | 100.00 |  |

**Endline**

| **FCM for anaemia in pregnancy is appealing to me.** | **Frequency** | **Percent** | **Cum.** |
| --- | --- | --- | --- |
| Neither agree nor disagree | 1 | 5.00 | 5.00 |
| Agree and Completely agree | 19 | 95.00 | 100.00 |
| Total | 20 | 100.00 |  |

1. **I like FCM for anaemia in pregnancy**

**Baseline**

| **I like FCM for anaemia in pregnancy** | **Frequency** | **Percent** | **Cum.** |
| --- | --- | --- | --- |
| Disagree | 1 | 5.00 | 5.00 |
| Neither agree nor disagree | 4 | 20.00 | 25.00 |
| Agree and Completely agree | 15 | 75.00 | 100.00 |
| Total | 20 | 100.00 |  |

**Endline**

| **I like FCM for anaemia in pregnancy** | **Frequency** | **Percent** | **Cum.** |
| --- | --- | --- | --- |
| Neither agree nor disagree | 2 | 10.00 | 10.00 |
| Agree and Completely agree | 18 | 90.00 | 100.00 |
| Total | 20 | 100.00 |  |

1. **I welcome FCM for anaemia in pregnancy**

**Baseline**

| **I welcome FCM for anaemia in pregnancy** | **Frequency** | **Percent** | **Cum.** |
| --- | --- | --- | --- |
| Agree and Completely agree | 20 | 100.00 | 100.00 |
| Total | 20 | 100.00 |  |

**Endline**

| **I welcome FCM for anaemia in pregnancy** | **Frequency** | **Percent** | **Cum.** |
| --- | --- | --- | --- |
| Agree and Completely agree | 20 | 100.00 | 100.00 |
| Total | 20 | 100.00 |  |

1. **Perception of SHPs on the feasibility of FCM**
2. **FCM for anaemia in pregnancy seems implementable**

**Baseline**

| **FCM for anaemia in pregnancy seems implementable** | **Frequency** | **Percent** | **Cum.** |
| --- | --- | --- | --- |
| Neither agree nor disagree | 1 | 5.00 | 5.00 |
| Agree and Completely agree | 19 | 95.00 | 100.00 |
| Total | 20 | 100.00 |  |

**Endline**

| **FCM for anaemia in pregnancy seems implementable** | **Frequency** | **Percent** | **Cum.** |
| --- | --- | --- | --- |
| Agree and Completely agree | 20 | 100.00 | 100.00 |
| Total | 20 | 100.00 |  |

1. **FCM for anaemia in pregnancy seems possible**

**Baseline**

| **FCM for anaemia in pregnancy seems possible** | **Frequency** | **Percent** | **Cum.** |
| --- | --- | --- | --- |
| Agree and Completely agree | 20 | 100.00 | 100.00 |
| Total | 20 | 100.00 |  |

**Endline**

| **FCM for anaemia in pregnancy seems possible** | **Frequency** | **Percent** | **Cum.** |
| --- | --- | --- | --- |
| Agree and Completely agree | 20 | 100.00 | 100.00 |
| Total | 20 | 100.00 |  |

1. **FCM for anaemia in pregnancy seems doable**

**Baseline**

| **FCM for anaemia in pregnancy seems doable** | **Frequency** | **Percent** | **Cum.** |
| --- | --- | --- | --- |
| Completely disagree | 1 | 5.00 | 5.00 |
| Agree and completely agree | 19 | 95.00 | 100.00 |
| Total | 20 | 100.00 |  |

**Endline**

| **FCM for anaemia in pregnancy seems doable** | **Frequency** | **Percent** | **Cum.** |
| --- | --- | --- | --- |
| Agree and Completely agree | 20 | 100.00 | 100.00 |
| Total | 20 | 100.00 |  |

1. **FCM for anaemia in pregnancy seems easy to use**

**Baseline**

| **FCM for anaemia in pregnancy seems easy to use** | **Frequency** | **Percent** | **Cum.** |
| --- | --- | --- | --- |
| Disagree | 2 | 10.00 | 10.00 |
| Neither agree nor disagree | 1 | 5.00 | 15.00 |
| Agree and Completely agree | 17 | 85.00 | 100.00 |
| Total | 20 | 100.00 |  |

**Endline**

| **FCM for anaemia in pregnancy seems easy to use** | **Frequency** | **Percent** | **Cum.** |
| --- | --- | --- | --- |
| Completely disagree | 2 | 10.00 | 10.00 |
| Agree and completely agree | 18 | 90.00 | 100.00 |
| Total | 20 | 100.00 |  |

1. **Comparison of the outcome measures scores by timepoint**

**Baseline**

| **Outcome** | **Timepoints** | **Observations** | **Mean (SD)** | **Min** | **Max** |
| --- | --- | --- | --- | --- | --- |
| **AIM composite score** | Baseline | 20 | 16.85 (2.00) | 13 | 20 |
|  | Endline | 20 | 18.50 (2.04) | 14 | 20 |
|  |  |  |  |  |  |
| **FIM composite score** | Baseline | 20 | 17.15 (2.32) | 13 | 20 |
|  | Endline | 20 | 18.25 (1.77) | 15 | 20 |
